# Negotiation of Calcified Canals

**DOI:** 10.3390/jcm13092703

**Published:** 2024-05-04

**Authors:** Antonis Chaniotis, Hugo Sousa Dias, Anastasia Chanioti

**Affiliations:** 1 Private Practice, 17676 Kallithea, Greece; 2 Private Practice, Dentistry Department, CESPU-IUCS University, 4585-116 Gandra, Portugal; hugosousadias@gmail.com; 3 School of Dentistry, National and Kapodistrian University of Athens (NKUA), 11527 Athens, Greece; anastasiachan88@gmail.com

**Keywords:** buckling resistance, calcification, free-hand approach, guided endodontics, pulp canal obliteration

## Abstract

The gradual formation of hard tissue along the root canal walls is a natural process associated with aging, typically progressing slowly over time. In reaction to tooth wear, operative procedures, vital pulp treatments, or regenerative endodontic procedures, hard tissue may also accumulate within the pulp canal space at a slow rate. In certain cases, such as dental trauma, autotransplantation, or orthodontic treatment, this deposition of hard tissue can accelerate unexpectedly, resulting in rapid narrowing or complete closure of the root canal space. This situation is called calcific metamorphosis (CM), root canal calcification, or pulp canal obliteration (PCO). Performing conventional endodontic therapy on severely calcified canals presents significant challenges and increases the risk of procedural accidents. Calcified canals introduce such complexity that dedicated negotiation concepts and specially designed instruments have been developed to deal with the challenge. This article seeks to review the existing methods for effectively navigating calcified canals and to introduce the buckling resistance activation test (BRAT) technique.

## 1. Introduction

In the ideal root canal negotiation scenario, where the canal space is patent from the canal orifice to the apical foramen, the challenge of root canal negotiation and enlargement is minimal. However, frequently, root canals might not be patent but fibrotic or obliterated by denticles, nodules, pulp stones, secondary or reactionary dentin formation, diffuse calcifications, or anatomical blockages that may prevent uneventful negotiation and instrumentation [1]. 

Efforts to perform root canal treatment in teeth with obstructed access due to calcific deposits often encounter challenges. The negotiation of small, calcified canals is considered challenging in all available difficulty assessment forms [2,3,4,5,6,7]. Attempting to identify the residual canal may necessitate the removal of the crucial dentinal structure, risking ledging, false canal creation, perforation, and weakening of the tooth. A higher number of instrument fractures and perforations are expected to occur in calcified root canals [8,9].

Root canal calcification, also known as calcific metamorphosis (CM) [10], refers to a pulpal reaction to trauma where there is rapid deposition of hard tissue within the canal space, leading to the obliteration of the root canal trajectory. This occurrence, alternatively referred to as dystrophic calcification, diffuse calcification, and calcific degeneration, depends on the severity of the trauma and the developmental stage of the root.

**Figure 1:**
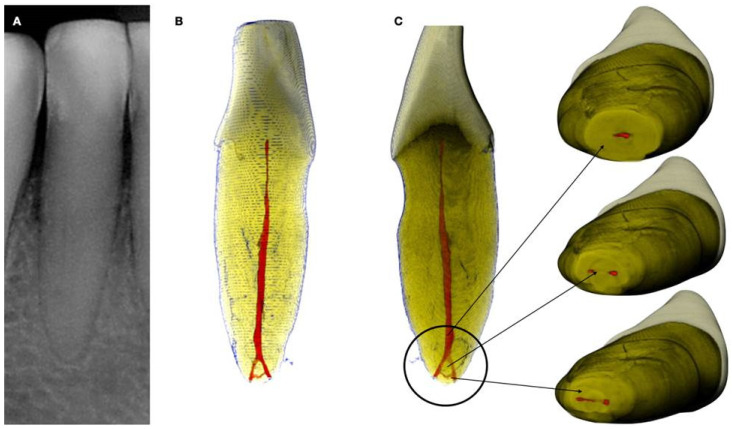
(**A**) Periapical radiograph of an abraded calcified mandibular incisor with no visible canal lumen. (**B**) Three-dimensional micro-ct reconstruction of the same calcified mandibular incisor that was extracted for prosthetic reasons. The micro-ct reveals a patent canal. (**C**) Apical root canal anatomy of the calcified canal lumen sectioned, revealing canal bifurcation and apical anastomosis (Skyscan 1172 micro-CT scanning device, Bruker MicroCT, Belgium) (images courtesy of Dr. Alexey Volokitin, city of Dnepr, Ukraine).

**Figure 2:**
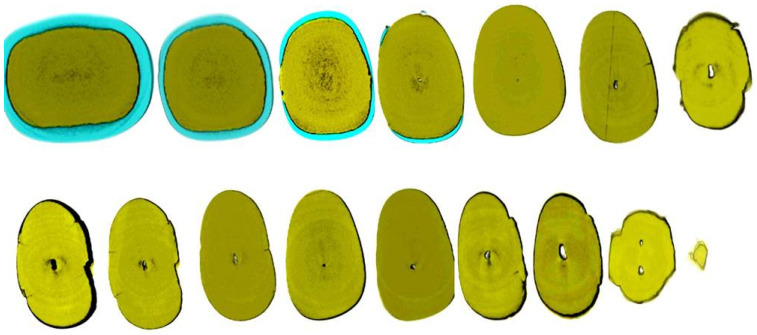
Axial micro-ct slices of the calcified mandibular incisor of Figure 1 reveal the cross-sectional root canal dimensions along the root. The crown of the tooth is completely blocked, and the canal is visible starting at the level of the CEJ. The root canal dimensions are constricted along the coronal part of the root, becoming wider in the middle, followed by an apical splitting into two canals. Calcified cases usually present an inverted taper (Skyscan 1172 micro-CT scanning device, Bruker MicroCT, Belgium) (images courtesy of Dr. Alexey Volokitin, city of Dnepr, Ukraine).

Radiographically, CM is categorized into partial calcification, where the pulp chamber is occluded, and the canal is constricted but detectable, and complete calcification, where both the pulp chamber and canal are hardly or not detectable. Despite the radiographic appearance, histological and micro-ct studies suggest the presence of a narrow canal, regardless of its visibility on radiographs (Figure 1 and Figure 2). 

**Figure 3:**
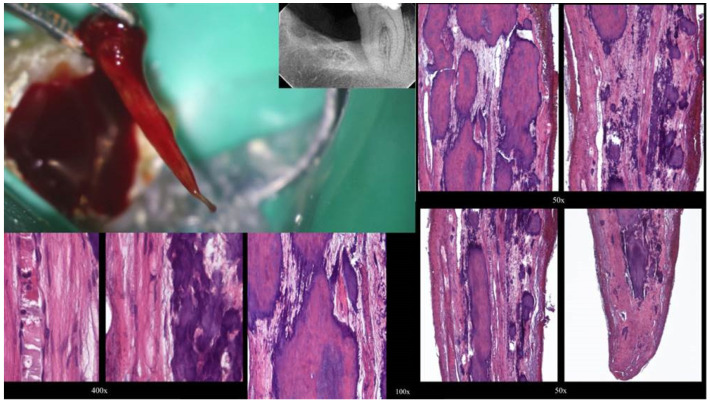
Histological images of the calcified vital pulp tissue that was removed during the root canal treatment of a second mandibular molar with deep periodontal distal lesion suffering from symptomatic irreversible pulpitis. Notice the linear calcified nodules formed along the root pulp vessels (hematoxylin–eosin staining) (clinical and radiographic images are courtesy of Dr. Chaniotis Antonis, and histological images are courtesy of Prof. Domenico Ricucci).

**Figure 4:**
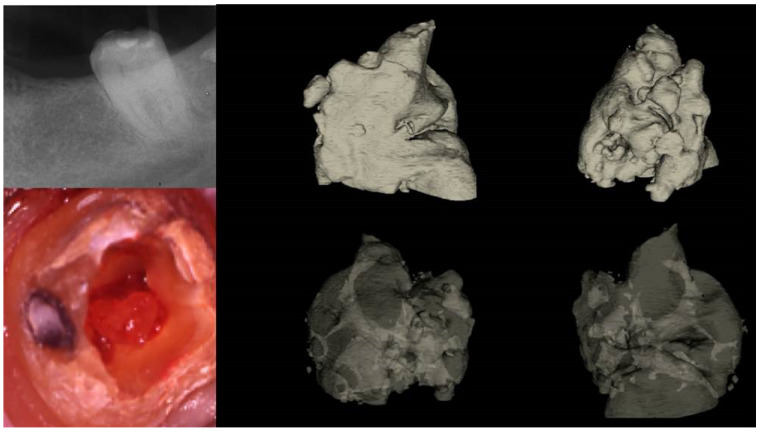
Removal of a pulp stone from a second mandibular molar suffering from irreversible pulpitis. Micro-ct evaluation of the external and internal structure of the pulp stone. Notice that the structure of the pulp stone is not solid, presenting an internal network of unmineralized tissue. The clinical significance is that the pulp stones can be dissected in smaller pieces and removed (clinical, radiographic, and micro-CT images courtesy of Dr. Chaniotis Antonis).

**Figure 5:**
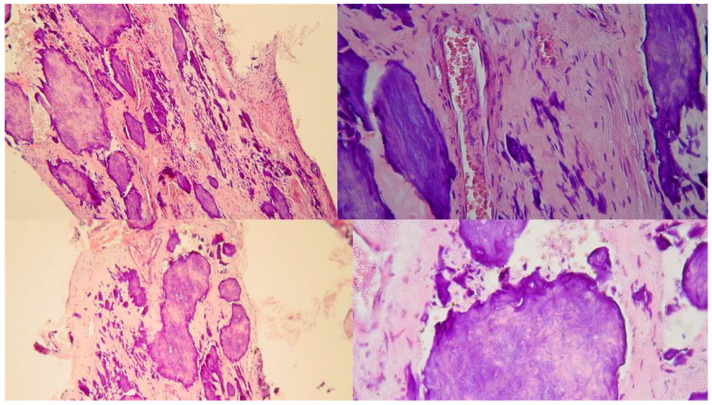
The free pulp stone’s histology shows distinct calcified nodules growing around a network of unmineralized extracellular matrix material and capillary vessels (hematoxylin–eosin staining) (histological images courtesy of Dr. Chaniotis Antonis).

Two distinct types of pulp calcification have been suggested to exist [11]: diffuse or linear calcifications predominantly found in the radicular pulp (Figure 3) and pulp stones (denticles) more common in the coronal region (Figure 4 and Figure 5).

The denticles, classified as true or false based on structure, exhibit morphological distinctions rather than chemical differences. True denticles closely resemble dentin, featuring dentinal tubules, odontoblastic processes, and a sparse presence of odontoblasts. In contrast, false denticles are characterized by concentric layers of calcified tissue enveloping a central cellular region, which could potentially be necrotic and serve as a focal point for denticle development. Based on location, denticles are categorized as embedded, interstitial, adherent, or free [11].

Complete obliteration of the root canal space is rare, and usually, it is accompanied by a thin filament of pulp tissue [12] (Figure 1). The partial obliteration, primarily affecting the pulp chamber, has minimal impact on the root canal and apical region. Localized obliteration, which can be attributed to traumatic incidents such as crown and root fractures, tooth displacement, jaw fractures, tooth re-implantation, and endodontic procedures, demonstrates a prevalence ranging from 3.8% to 24% [12]. On the other hand, generalized obliteration, because of aging, manifests in older individuals and may lead to complete obliteration or the presence of a hairline-thin pulp chamber. This condition is frequently associated with attrition, deep dental caries, and extensive dental restorations [13]. Pulp canal obliteration is reported to occur in 69–73% of incisors affected by root fractures [14]. Pulp stones and pulp obliteration were commonly observed in older individuals but notably prevalent even among younger adults diagnosed with Marfan syndrome [15,16]. Treating calcified canals presents extreme challenges due to the narrowed pulp chamber and canal lumens, complicating access location, negotiation, cleaning, and shaping procedures. This paper aims to review the available treatment options for the management of calcified root canals, suggest useful clinical aids, and introduce a new technique for calcified canal negotiation. 

## 2. Radiographic Interpretation of Root Canal Calcification

The radiograph plays a crucial role in root canal therapy, aiding not only in diagnosis but also in evaluating several factors, including curvature, size, number, shape, and degree of calcification of pulp canals. However, the apparent width of a canal on a periapical radiograph may not always accurately reflect its true clinical size. For example, a canal that appears clear on a radiograph might pose challenges in locating or navigating with endodontic instruments. Conversely, a canal that appears calcified on the radiograph may still be manageable clinically. Comparing the radiographic appearance of root canal sizes to their actual diameter, it was shown that while all specimens had canals histologically, seven roots exhibited no visible canal space radiographically [17]. This finding challenges the assumption that canals may completely calcify, suggesting that even if a canal appears fully calcified on the radiograph, there may still be sufficient tissue within it to cause endodontic problems if that tissue becomes necrotic or inflamed. The absence of visible canals could stem from various factors such as complex regional anatomy, canal calcification, or the division of a single canal into smaller ones [17]. Additionally, in some cases, no apparent explanations for the lack of visibility exist, suggesting that primary and secondary dentin thickness could obscure small canal spaces. It is crucial to note that despite radiographic visibility, the actual dimensions of canals may vary significantly, leading to challenges in treatment. These discrepancies underscore the importance of clinical judgment and meticulous treatment planning in root canal therapy [17].

## 3. What Causes These Differences?

It is plausible that what appears as a canal space may entirely or partially result from variations in the mineralization of dentin surrounding the pulp. Essentially, it could be theorized that secondary dentin, whether regular or irregular, developed on the canal walls, exhibiting significantly lower radiodensity compared to primary dentin. This would create a radiographic impression of a narrower canal diameter [17]. Complete radiographic obliteration of the pulp space does not necessarily indicate the absence of a canal. In most cases, the sensitivity of conventional radiographs is insufficient to render calcified canal images discernible [18,19]. 

## 4. CBCT Identification of Calcified Canals

Several recent articles have highlighted advancements in technology regarding the utilization of CBCT for identifying and managing calcified canals [20,21,22]. These advancements include offering supplementary information to aid clinicians in locating canals or utilizing the data to create a stent for precise alignment and depth control of the bur. Multiple studies [20,21,22,23] have demonstrated CBCT’s superior sensitivity in canal detection compared to conventional radiography. Evaluating the use of CBCT for the detection of root canals [23], it was specifically found that endodontists missed identifying root canals in 40% of cases when compared to CBCT. However, there was limited evidence regarding CBCT detection rates concerning canal diameter. The influence of CBCT voxel size on canal detection has been proposed, with smaller voxel sizes theoretically providing higher resolution for detecting fine structures like constricted root canals. Yet, it was suggested that examiner experience may outweigh voxel size in diagnostic reproducibility [24]. Additionally, the choice of CBCT view size impacts resolution, with smaller fields of view offering greater resolution due to the smaller area being projected over the detector. Optimizing CBCT exposure is crucial to balance dose reduction while maximizing diagnostic yield, particularly in cases requiring identification of calcified canal systems. Higher resolution, achieved through smaller field-of-view images, is essential for such cases. However, resolution alone does not dictate canal identification; contrast plays a significant role. Lowering the kV improves contrast but increases the noise-to-signal ratio, necessitating a balance between spatial resolution, contrast, and noise for effective detection. CBCT provides valuable depth measurements for canal detection, facilitating predictable access during treatment. If a canal is not found at the expected depth, clinicians can adjust their search laterally, streamlining the access procedure and reducing treatment time [25].

## 5. Anatomy of the Calcified Canal

The volume of the pulp space decreases over time due to the deposition of regular secondary dentin. Additionally, reactionary and reparative dentin (previously categorized together as tertiary or irritation dentin) further diminishes the pulp space. These forms of dentin are laid down to decrease the porosity of dentinal tubules, which may be exposed to the oral environment due to factors such as caries, trauma, or dental procedures or to heal direct pulpal exposures. This process is most frequently observed in pulp horns and on the floor and roof of the pulp chamber in molars, which can transition from a large rectangular cavity in youth to a flattened disc in old age [26,27]. In anterior teeth, the pulp gradually recedes in a cervical direction, becoming narrower and sometimes leaving no soft tissue within the crown at all [28]. Calcified tissue deposition often occurs concentrically towards the center, particularly in the coronal portions of the canal system, while deeper regions of the root canals tend to remain widely patent even into advanced age (Figure 1 and Figure 2). Cumulative injuries to the pulp result in decreased vascularity and cell content, accompanied by an increase in fibrosis, primarily affecting the coronal aspects of the canal system where external irritants exert the greatest influence [26].

Pulp fibrosis or atrophy is typically considered a histological alteration that may not be clinically discernible unless the pulp space is accessed, questioning its diagnostic value. Conversely, pulp calcification is often clinically detectable before treatment and can directly impact treatment prognosis. Calcification alone does not necessarily indicate progressive inflammation or necrosis of the pulp. Traumatically induced calcified pulps result in pulp necrosis in fewer than 7% of cases [29,30]. 

**Figure 6:**
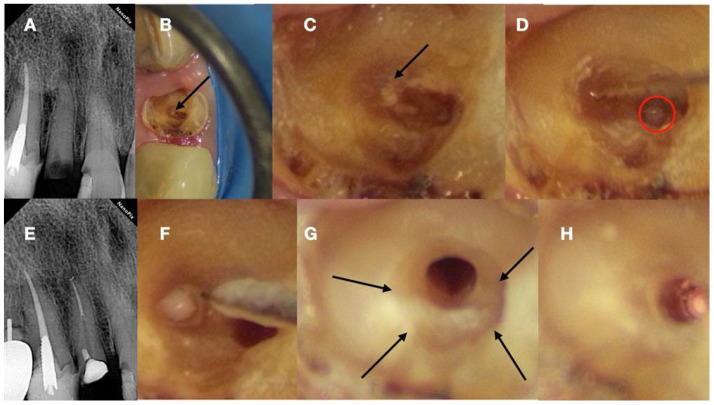
(**A**) Preoperative radiograph of a calcified lateral incisor that suffered a crown fracture. The root canal treatment negotiation was initiated but stopped because of the non-detectable canal and misorientation. The root canal is not visible in the radiograph. (**B**) Clinical view of the access cavity after cleaning the debris. The arrow points to a white spot, indicating a possible canal. (**C**) Higher magnification clinical view. The peripherical dentine is yellow, followed by a central circular grey area that holds a white spot of accumulated debris in the white spot (black arrow). (**D**) A D-finder file (Mani, Japan) negotiating the calcified canal (initial catch). The red circle indicates the arrested misoriented previous access. (**E**) Postoperative radiograph. (**F**) Clinical view of the initial glide path file removing pieces of coronal restrictive dentin. (**G**) Clinical view of the calcified canal after the shaping procedures. Transillumination reveals the radial orientation of the dentinal tubules. The reflection of the microscope light gives the characteristic butterfly effect impression (arrows). (**H**) The clinical view of the gutta-percha cut deep inside the canal during post-space preparation (clinical and radiographic images courtesy of Dr. Chaniotis Antonis).

**Figure 7:**
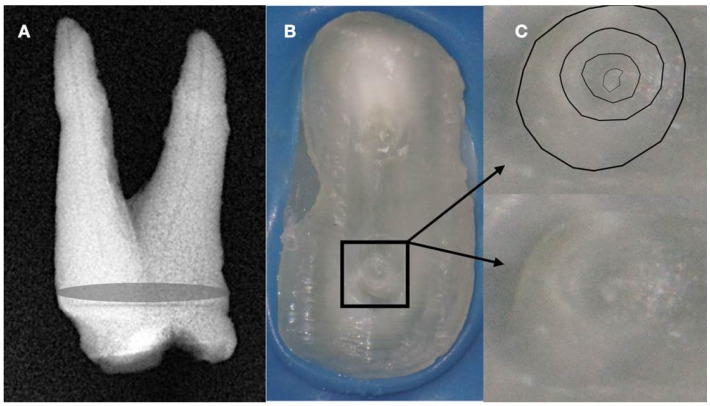
(**A**) Radiographic image of an extracted partially calcified maxillary premolar. (**B**) Microscopic view of the cross-section of the calcified orifices of the extracted maxillary premolar. (**C**) Magnified microscope view of the area in the square. Notice the circular deposits of calcified material in the periphery reflecting different stages of tertiary, reactionary, or reparative dentine formation. Concentrating on these circular deposits, a grey area exists with a white spot of debris accumulation in the center. This white spot is indicative of the canal’s location (clinical and radiographic images courtesy of Dr. Chaniotis Antonis).

Furthermore, diffuse calcifications typically form as amorphous, unorganized linear columns aligned parallel to the blood vessels within the pulp, resembling calcifications observed in other degenerating tissues in the body (Figure 3). Fibrillar calcifications represent one aspect of the regressive changes experienced by the pulp. The prevalence of pulp calcifications appears to be relatively high, particularly under microscopic examination, as many calcifications are too small to be discernible on radiographs. Calcifications tend to increase with age, with approximately 90% of individuals aged 50 years or older reported to be affected [13]. As teeth age, there is a decline in vascular, lymphatic, and nerve supplies, along with a decrease in the size and number of fibroblasts [13]. Notably, a reduction of 15.6% in crown odontoblasts, a decrease of 40.6% in root odontoblasts, and diminished secretory activity have been observed, indicating the compromised reparative capacity of the pulp with aging [27]. Age-related changes include increases in cross-linkages and the number of collagen fibers, lipid infiltration, and calcifications [27]. Despite numerous studies investigating the mechanisms underlying these age-related changes, much remains to be understood regarding the biological control mechanisms responsible for cellular activity and survival throughout life. The mechanism of hard tissue formation during calcific metamorphosis (CM) remains unclear, although several hypotheses have been proposed. It was hypothesized that hard tissue deposition occurs either due to stimulation of pre-existing odontoblasts or loss of their regulatory mechanism [19]. Conversely, CM was described as a response to severe injury to the neurovascular supply of the pulp, which, upon healing, leads to accelerated dentin deposition and is closely related to the loss and re-establishment of the pulpal neural supply [31]. Neither mechanism has been conclusively proven nor studied, necessitating further investigation to provide an evidence-based understanding of this phenomenon. Calcific metamorphosis is characterized by the formation of osteoid-like tissue produced by odontoblasts at the periphery of the pulp space or by undifferentiated pulpal cells undergoing differentiation due to traumatic injury [28,32]. This results in the simultaneous deposition of dentin-like tissue along the periphery of the pulp space (root canal walls) and within the pulp space proper (root canal). Over time, these tissues can fuse, resulting in a radiographic appearance of a root canal space that has become rapidly and completely calcified [33]. Clinically, there is usually a clear distinction between the irregular, calcified tissue and the peripheral dentinal walls, reflecting the distinct histological characteristics and mechanisms of tissue formation obliterating the pulp space. Under magnification and illumination provided by a dental operating microscope, these tissues reflect the light in different ways. Typically, when negotiating a calcified canal under a dental operating microscope, the operator evaluates the various colors and textures of dentin on the axial view plane. In a single-rooted calcified tooth, the concentric axial orientation of dentinal tubules is visible under the microscope, presenting a concentric radial appearance (Figure 6 and Figure 7). If not readily visible, transillumination from a different angle can enhance intracanal visibility (Figure 6).

This concentric radial appearance points to a greyer area in the center that demarcates the area of secondary and reactionary dentine formation, occluding the canal space. This irregular dentine gives a different reflection under the microscope with a more greyish appearance. The thin canal space usually lies in the center of the irregular calcified tissue. Because of the debris production during deep troughing with burs or ultrasonics, a white spot is created over the canal space dimple, demarcating the calcified canal orifice (Figure 6 and Figure 7). 

Determining the position of calcified pulp chambers and locating calcified root canal entrances in multirooted teeth presents even greater challenges. Based on an anatomical study of 500 teeth, several principles have been proposed to aid in this determination [34]:

- Law of centrality: The pulp chamber floor consistently lies at the center of the tooth, aligned with the cemento-enamel junction (CEJ).
- Law of concentricity: The pulp chamber walls maintain concentricity with the external tooth surface at the CEJ level, meaning the external root surface mirrors the internal pulp chamber anatomy.
- Law of the CEJ: The distance from the clinical crown’s external surface to the pulp chamber wall remains consistent around the tooth’s circumference at the CEJ level.
- Law of symmetry 1: Except for maxillary molars, canal orifices are equidistant from a line drawn mesially to distally through the pulp chamber floor.
- Law of symmetry 2: Except for maxillary molars, canal orifices lie on a line perpendicular to a mesial-distal line across the center of the pulp chamber floor.
- Law of color change: The pulp chamber floor appears darker than the walls.
- Law of orifice location 1: Root canal orifices are located at the junction of the walls and the floor.
- Law of orifice location 2: Root canal orifices are found at the angles of the floor-wall junction.
- Law of orifice location 3: Root canal orifices are situated at the terminus of the root developmental fusion lines.

In calcified pulp chambers, identifying the floor-wall junction and root developmental fusion lines can be challenging. Ultrasonic removal of loosely attached calcified structures can expose developmental fusion lines leading to canal orifices. If the calcified tissue is firmly attached, the anatomy may be altered, hiding the developmental fusion lines. Canal orifices can be found by progressively excavating calcifications following orifice location laws. Deep troughing with ultrasonics or long-shafted burs can reveal the floor-wall junction angles and developmental fusion lines, where calcified canal orifices are often located.

In maxillary molars, searching for the floor–wall junction angles and the dark developmental lines outlining the chamber perimeter aids better in orifice detection. Mandibular molars and premolars rely mostly on developmental fusion lines on the pulp floor for guidance. Different colorations within the pulp floor are recognized cues for locating canals, with variations in hydration affecting color perception. Moistening the dentin enhances the contrast between grey pulpal floor shades and white secondary dentin shades, aiding in canal identification [25]. After identifying and removing the calcified tissues, the root developmental fusion lines and the floor wall junction become visible. Finding the canal orifice is the first step in negotiating inside a calcified canal. 

## 6. Negotiation of Calcified Canals

Typically, navigating the challenging calcified spaces requires the use of small files for initial pathfinding. However, these files often lack the necessary rigidity to traverse narrow passages and may bend or break when subjected to vertical watch-winding strokes. Consequently, this can result in damaged files without successful negotiation through the calcified tissue. An alternative approach involves employing size 8 and 10 K-files interchangeably, employing a gentle watch-winding motion with minimal vertical pressure, and regularly replacing the instruments before they become fatigued [35]. Additionally, a specialized technique utilizing K-files with modified tips has been developed to penetrate constricted canals effectively. In this technique, the tip of a #10 K-file is diagonally sliced to enhance its thinness. This modified K-file, characterized by a finely tapered tip and appropriate stiffness, demonstrates a high penetration potential, facilitating effective traversal through narrow or calcified canals [36].

Recently, various specially designed ‘pathfinding’ instruments were introduced for use in difficult negotiation scenarios. These instruments are manufactured by special work-hardened or fiber-reinforced stainless steel alloy and have various designs to enhance their rigidity and buckling resistance. Some of these glide finder files have modified tips, tapers, and cross-sectional designs to penetrate through anatomical impediments and calcified structures. Instruments characterized by a reduced flute design, such as the Canal Pathfinder (JS Dental, Ridgefield, CT, USA), or those featuring enhanced shaft strength like the Pathfinder CS^TM^ (Kerr Manufacturing Co., Romulus, MI, USA), demonstrate improved efficacy in penetrating highly calcified root canals. C + Files (Dentsply, Tulsa, OK, USA) are suggested to be advantageous for the initial instrumentation of calcified root canals, incorporating a cutting tip for engaging dentin. D-finder files (Mani, Japan) can be used effectively to penetrate through fibrotic or calcified canal pathways. They are manufactured with special 18-8 hard fiber stainless steel, making the files stiffer than K-files, and have a special design with a D-cross sectional shape, fewer flutes, and a non-cutting tip offering great negotiation potential. Very recently, variable regressive taper glide finder files with active tips (Mani, Japan) were also introduced for the catheterization of calcified canals. The validity of all these glide finder files must be evaluated.

In addition to the glide finder files, various long-shafted burs were introduced for deep troughing along the long axis of the obliterated root. Long-neck round burs (LN-bur), such as those from Caulk/Dentsply (Tulsa, OK, USA), and extended-shank round burs, like the Mueller bur (Brasseler, Savannah, GA, USA), facilitate the identification of orifices in calcified canals. Furthermore, the Munce Discovery bur (CJM Engineering, Santa Barbara, CA, USA), akin to the Mueller bur but distinguished by a stiffer shaft and available in smaller head sizes, presents an alternative option. The extended shank of these burs strategically positions the handpiece away from the tooth, enhancing the clinician’s visibility during this intricate procedure. The long-shafted Munce burs are used in a crown-down approach of 1 mm increments. Care should be taken not to deviate from the long axis of the root. Multiple radiographs might be needed for the continuous control of the tooth’s long axis. Deep troughing with burs inside the root to find the calcified canal trajectory is always challenging and might result in many complications when attempted without guidance. The undesirable complications range from file breakage, ledging, and false canal creation to more severe perforation damage or removal of excessive tooth structure, rendering the obliterated tooth non-restorable. Alternative to the long-shafted burs, ultrasonic tips are also recommended for the detection and negotiation of obliterated canals, especially when working under magnification. They offer enhanced visibility under the DOM, and they can be used in conjunction with methylene blue dye for very detailed troughing of root dentin. Long-shafted burs and ultrasonic tips can be used safely and effectively in the coronal and middle third of the calcified canals. For more apical penetration, a technique called buckling resistance activation test (BRAT) can be used.

## 7. Buckling Resistance Activation Test (BRAT) Negotiation of Calcified Canals

Buckling occurs when an instrument experiences sudden sideways deflection due to compressive force overpowering its resistance. Instruments with low buckling resistance are prone to deformation and may struggle to penetrate deeply into calcified canals. Proper buckling resistance facilitates both the exploration of canal orifices and the negotiation of narrow canal walls. However, overly stiff instruments with strong buckling resistance may lead to canal aberrations, such as ledges and perforations during negotiation, potentially compromising clinical outcomes. Negotiating through the tiny, constricted calcified canal presents considerable challenges, even with the utilization of small stainless-steel glide finder files [37]. Recently, small-size NiTi engine-driven instruments have been introduced specifically for glide path preparation, but their use is limited to patent canal trajectories. In calcified and sclerotic canals, small-size NiTi instruments with non-cutting tips are prone to torsional failure when attempting to penetrate through calcifications. When a canal trajectory is larger than the engine-driven glidepath tip, a non-cutting tip is beneficial to avoid transportation. For canals smaller than the glidepath tip, like calcified canal trajectories, a cutting tip is believed to allow easier and safer penetration [38]. 

**Figure 8:**
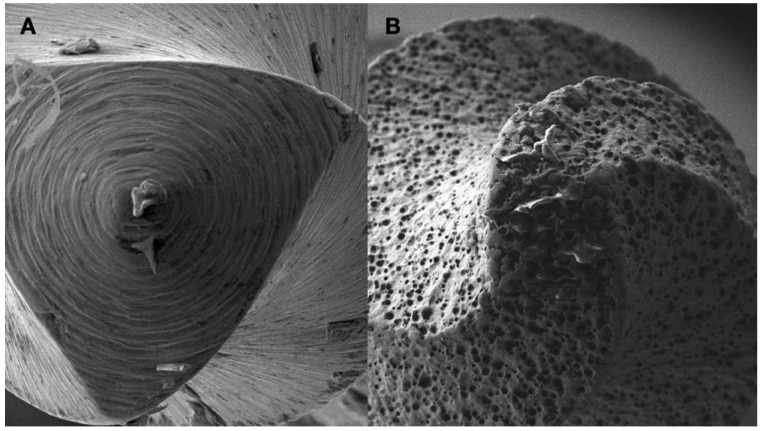
(**A**) Scanning Environmental Microscope image of the tip of a Grinded Niti file. (**B**) Scanning Environmental Microscope image of the tip of an Electrical Discharge Machined Niti file. The EDM procedure creates a rough surface in the tip of the instrument, making the tip active when activated and increasing the cutting efficiency of the file (images courtesy of Dr. Alexey Volokitin, city of Dnepr, Ukraine).

Recently, electrical discharge machining (EDM) has been utilized for the manufacturing of endodontic files, altering the molecular structure of the file’s surface to enhance cutting efficiency while maintaining flexibility [38] (Figure 8). The resulting rough surface of EDM file tips exhibits increased cutting efficiency compared to the conventional ground file tips when the file is activated against a calcification blockage (Figure 8). 

**Figure 9:**
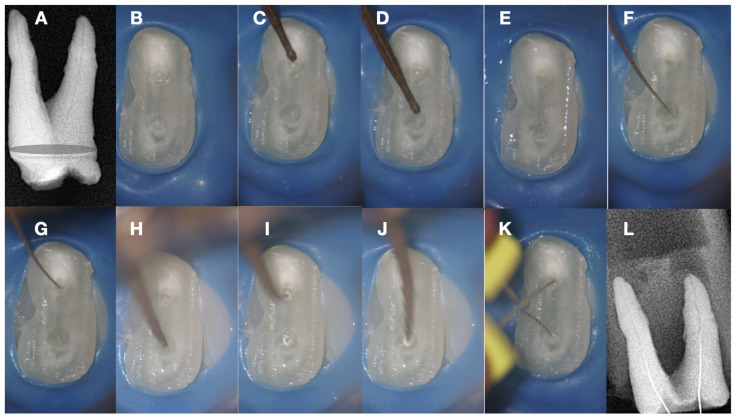
(**A**) Radiographic image of an extracted partially calcified maxillary premolar. (**B**) Magnified view at the level of the axial cut (a grey area in a). (**C**,**D**) Creation of initial access dimple in the white spot indicating the calcified orifice. (**E**) Continuous chelation of the cut dentinal surface (**F**,**G**). An initial unsuccessful attempt to negotiate the calcified canal orifices with D-finder files iso 08. (**H**) Fitting of the tip of an EDM file (Hyflex EDM-one file, Coltene) in the dimple without activation. (**I**,**J**) On-spot buckling resistance activation test (BRAT) negotiation of the EDM files. (**K**) Negotiation of the 08 d-finders inside the calcified canals after the BRAT negotiation technique removed coronal calcified canal obstructions. (**L**) Radiographic verification of calcified canal negotiation (images and radiographs courtesy of Dr. Chaniotis Antonis).

Whenever the rough spark eroded EDM tip is activated against the calcified tissue, it can carve its way through, creating an initial dimple in the calcified canal orifice (Figure 9H–I). The carving of the pilot dimple can be further facilitated by using a continuous chelation irrigation technique (Figure 9E). 

**Figure 10:**
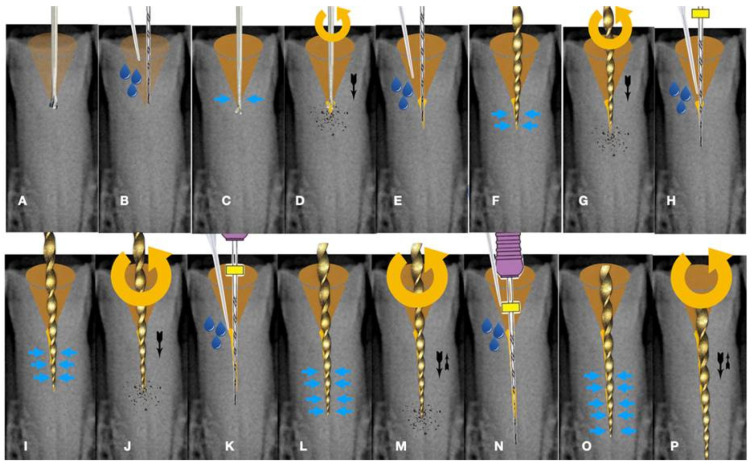
Buckling resistance activation test (BRAT) negotiation of calcified canals graphic explanation (**A**). Long-shafted bur access cavity preparation (**B**). Continuous chelation concept irrigation and D-finder negotiation test (**C**). Long-shafted access bur of smaller diameter insertion without activation (tactile feedback) (**D**). Single-stroke activation of the bur (Tactile Controlled Activation) (**E**). Continuous chelation concept irrigation and D-finder negotiation test. If there is no negotiation, continue to the next step (**F**). EDM or Active tip file placement inside the access dimple without activation (**G**). Activation of the EDM file at 500 rpm/3 NCM and application of vertical axial pressure against the calcified canal entrance (long press release twice) (**H**). Continuous chelation concept irrigation and D-finder negotiation test. If there is no negotiation, continue to the next step (**I**). EDM or Active tip file placement deeper inside the dedicated access dimple without activation (**J**). Activation of the EDM file at 500 rpm/3 NCM and application of vertical axial pressure against the calcified canal entrance (long press release twice). As the file goes deeper inside the root, the buckling resistance of the file increases because the lateral walls will not allow the file to buckle and succumb. The axial pressure is transported to the file tip, allowing the file to penetrate (**K**). Continuous chelation concept irrigation and D-finder negotiation test. If there is no negotiation, continue to the next step (**L**). EDM or Active tip file placement deeper inside the dedicated access dimple without activation (**M**). Activation of the EDM file at 500 rpm/3 NCM and application of vertical axial pressure against the calcified canal entrance (long press release twice) (**N**). Continuous chelation concept irrigation and D-finder negotiation test (**O**,**P**). If there is no negotiation, repeat the previous steps. Horizontal light blue arrows: file is engaged in the lateral canal walls, vertical black arrows: direction and force of file movement, Blue drops: irrigation.

The initial dimple is deepened progressively in a crown-down sequence until a patent pathway is met and confirmed (Figure 9 and Figure 10). 

As the instrument goes deeper inside the blocked root canal, the buckling resistance of the instrument increases because the lateral canal walls will not allow the instrument to buckle. Any axial pressure applied will be transported to the file tip. 

Buckling resistance activation test (BRAT) negotiation is defined as the activation of an engine-driven file with an active or dynamic tip against the conditioned calcified tissue that is blocking the canal trajectory (Figure 9 and Figure 10). The tip of the engine-driven instrument is attached to the calcification point without activation. The instrument is activated according to the manufacturer’s instructions, and the file is pushed in an axial direction against the calcification (long press and release). The lateral buckling movement of the file is restrained by the canal walls. When the progression of the file is achieved, the file is removed from the canal, and the canal is checked for patency with a hand file. If patency is achieved, then standard root canal treatment procedures can be continued. If there is no patency, the same procedure can be attempted deeper inside the calcification. The step-by-step progression of a rotary file through the calcification with the BRAT technique is described in Figure 10. 

**Figure 11:**
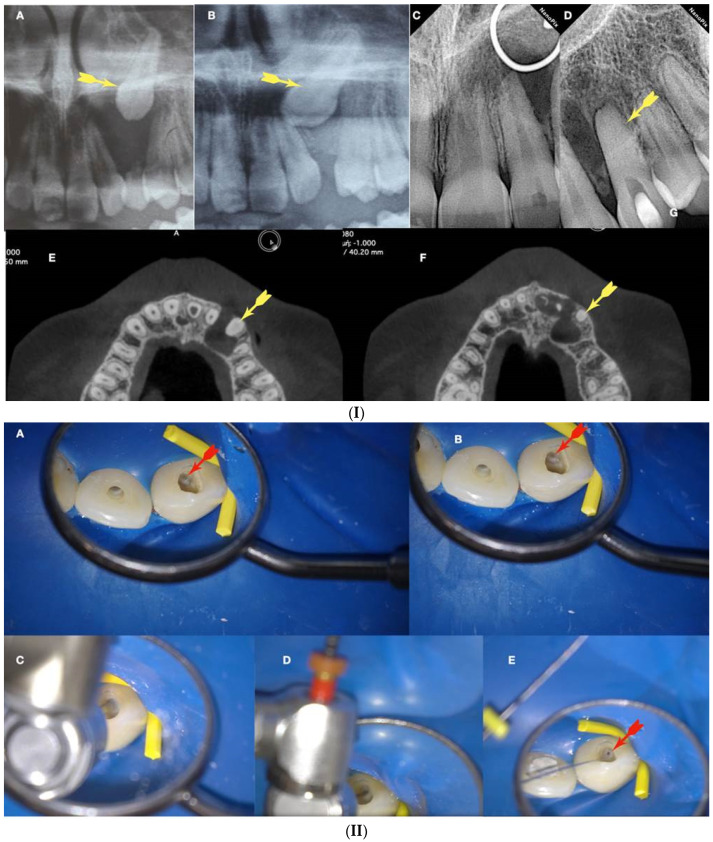

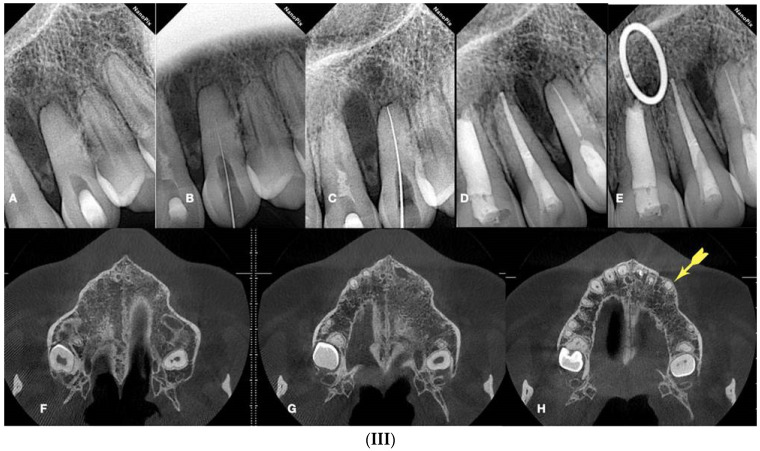
(**I**) (**A**) Preoperative panorax focused on the impacted right maxillary canine, revealing a large lesion. (**B**) Two-year follow-up panorax after the surgical removal of the lesion revealing healing. (**C**,**D**) Periapical radiographs reveal recurrent disease 6 years after the surgical intervention. Notice that the maxillary canine was brought in occlusion with orthodontic treatment, but the canal was rendered radiographically blocked. (**E**,**F**) CBCT axial slices evaluate the magnitude of the periapical lesion and the root canal calcification of the maxillary canine. (**II**) (**A**,**B**) Clinical microscopic image from two different angles of the previously initiated access cavities. Notice the gray spot indicating the calcified root canal orifice (yellow arrow). (**C**) Clinical image of the initial troughing of the calcified canal with a round long shafted bur (Munce bur). (**D**) EDM file during the buckling resistance activation test negotiation technique. (**E**) Clinical image of the calcified orifice after the first BRAT stroke. Canal negotiation with the 08 D-finder file is now possible. (**III**) **A** Preoperative periapical radiograph. (**B**) Periapical radiograph demonstrating the initial negotiation of an 08 D-finder after the first BRAT stroke. (**C**) Periapical length determination radiograph. (**D**) Postoperative radiograph. (**E**) One-year follow-up periapical radiograph. (**F**–**H**) Three-year follow-up cbct reveals complete healing of the periapical lesion (axial slices) (clinical images and radiographs courtesy of Dr. Chaniotis Antonis). Red arrows indicate the calcified canal location, and Yellow arrows indicate the impacted canine. The white circle is the projection of a nose piercing ring in the radiograph (not a symbol).

**Figure 12:**
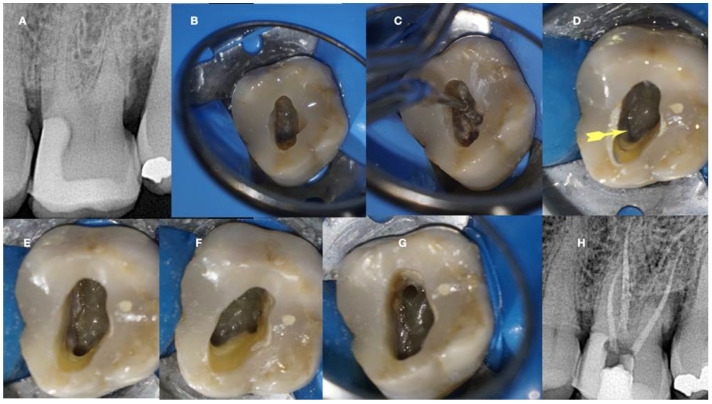
Calcified maxillary molar negotiation (**A**). Preoperative radiograph of the maxillary molar with no detectable canals (**B**). A clinical view of the access cavity reveals a completely calcified chamber (**C**). Clinical view of the ultrasonic troughing of the pulp floor. Notice that the pulp floor color is grey (**D**). Clinical view of the axial wall floor junction developmental line (yellow arrow). The canal entrances in a maxillary molar always originate from this line (**E**–**G**). Clinical image of the shaped canals. Notice that the location of the canals in a calcified maxillary molar is always lying along the wall–floor junction developmental lines (**H**). Postoperative radiograph of the calcified maxillary molar treatment (clinical images and radiographs courtesy of Dr. Chaniotis Antonis).

**Figure 13:**
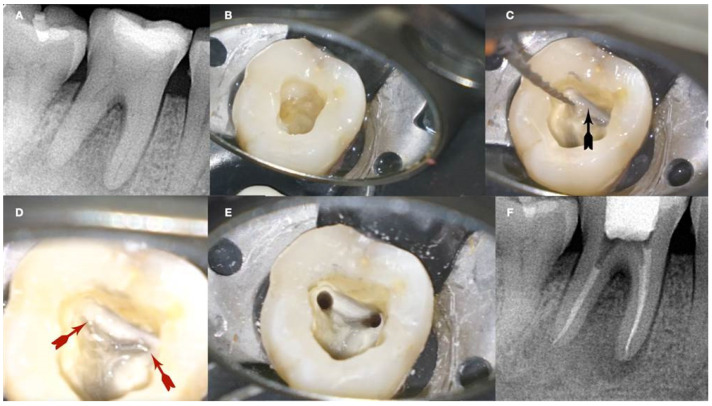
Calcified mandibular molar negotiation (**A**). Preoperative radiograph of a calcified mandibular molar with no visible canals (**B**). Clinical view of the calcified chamber (**C**). Clinical view of the calcified tissue blocking the access to the mesial canal system. Notice the white color of the calcification. The axial walls are yellow, and the pulp floor is always grey (**D**). Clinical view of the pulp floor developmental lines after the removal of the white calcified tissue. Chasing the dark grey developmental lines to their termini will reveal the calcified canal entrances (**E**). Clinical view of the canals shaped (**F**). Postoperative radiograph of the calcified mandibular molar canal treatment (clinical images and radiographs courtesy of Dr. Chaniotis Antonis). Black arrow: calcified structure covering the canal orifices and the developmental pulp chamber lines, red arrows indicate the canal orifices after the removal of the white calcified structure (black arrow).

**Figure 14:**
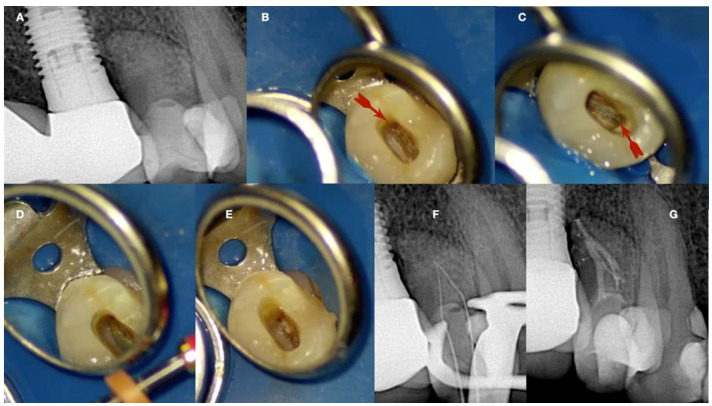
Calcified maxillary premolar negotiation (**A**). Preoperative radiograph of a calcified maxillary premolar associated with apical periodontitis (**B**). Clinical view of the palatal calcified canal orifice (red arrow) (**C**). Clinical view of the buccal calcified canal orifice after chasing the pulp floor developmental line to its far end (red arrow) (**D**). Clinical view of the EDM file buckling resistance activation test negotiation (**E**,**F**). Clinical and radiographic images of the calcified canal negotiation, (**G**) postoperative radiograph of the calcified maxillary premolar treatment (clinical images and radiographs are courtesy of Dr. Chaniotis Antonis).

In Figure 11, Figure 12, Figure 13 and Figure 14, initial negotiation through the calcified canal orifices was carried out using the BRAT technique until canal patency was met. The conditioning of the calcified tissue blocking the canals for the BRAT technique to be effective is preferably accomplished with the continuous chelation concept.

## 8. The Continuous Chelation Concept

Pulp calcifications consist of both organic and inorganic components. The calcium present in hydroxyapatite (Ca_3_(PO_4_)_5_OH) crystals is one of the main inorganic elements of pulp calcifications. For penetration through the calcifications, chelating agents are chosen due to their direct action on calcium ions. Any change in the calcium ratio can significantly alter the original proportion of organic and inorganic components, which can alter the irregular calcification of tissue permeability, microhardness, and solubility [39]. During calcified canal penetration, altering the calcified substrate is of great importance and can be achieved with chelators [40].

Calcified material is a substrate with a complex organic and inorganic structure. It is well known that NaOCl is a non-specific proteolytic agent that can remove organic material, as well as magnesium and carbonate ions [41]. Thus, NaOCl fragments long peptide chains and chlorinates protein terminal groups. Consequently, hypochlorite solutions may affect the mechanical properties of the calcified substrate via the degradation of organic components. The mechanical properties, such as microhardness, roughness, elastic modulus, flexural, and fatigue strength, can be influenced by treatment with NaOCl. Significant changes in hardness following NaOCl treatment indicate the potent direct effects of this chemical agent on the organic and mineral content of calcified structures. Moreover, the volumetric contraction of NaOCl-treated dentine and changes in the crystallinity of dentine apatite are important factors in determining the intrinsic hardness profile of the calcification. 

Continuous chelation is the concept of using a single mix of a weak chelator with NaOCL throughout the entire root canal preparation procedure without causing a reduction in the antimicrobial and proteolytic activity of NaOCL [42]. Etidronic acid, also known as “1-Hydroxyethylidene-1, 1-Bisphosphonate” HEBP, or HEDP, is a soft biocompatible chelator utilized in direct combination with sodium hypochlorite to form an all-in-one deproteinizing, disinfecting, and chelating solution. It is the only chelator available as a certified commercial product, “Dual Rinse HEDP”, approved for endodontic usage.

Combining a weak chelator with NaOCL solution, a single irrigation solution mixture with soft tissue dissolving ability and antibacterial properties with chelating capability can be created, which can be considered a good alternative to the conventional irrigation protocol (sequential irrigation) with NaOCL followed by using a strong chelator such as EDTA. The obvious benefit is that only one solution is required for root canal cleansing and decontamination, decreasing the time for irrigation and providing better conditioning of root canal walls. The continuous chelation concept will condition the calcification blocking the canal so that the endodontic instruments can penetrate before they buckle and fail. The BRAT negotiation technique is facilitated with the continuous chelation concept without risking the deleterious effects that strong chelators have on the canal walls. Continuous chelation can minimize the risk of false canal creation during the BRAT negotiation technique.

## 9. Guided Negotiation of Calcified Canals

Even with adherence to all the previous recommendations, locating calcified root canals remains a challenging and time-consuming task, often resulting in significant loss of tooth structure. This loss is associated with a heightened risk of fracture and perforation, ultimately compromising the tooth’s prognosis [43,44]. The failure rate of treatment for pulp canal obliteration (PCO) has been reported to range from 20% to 70%, influenced by the clinical expertise and anatomical knowledge of the operator, as well as the information provided by two-dimensional (2D) and three-dimensional (3D) radiographic examinations [45,46]. While it is established that highly experienced endodontists can achieve favorable outcomes, even with the assistance of a dental operating microscope (DOM), long-necked burs, and ultrasonic tips, the process of achieving an adequate access cavity and locating the root canal may still result in excessive loss of tooth structure and an increased risk of fracture and perforation [20,44,47,48]. A novel clinical approach for managing teeth with PCO has been introduced, termed “Guided Endodontics”. Guided endodontics, employing either static-guided (SG) or dynamic-guided (DG) techniques, has emerged as an alternative for access cavity preparation in the clinical management of complex cases [49].

## 10. Static-Guided Technique

**Figure 15:**
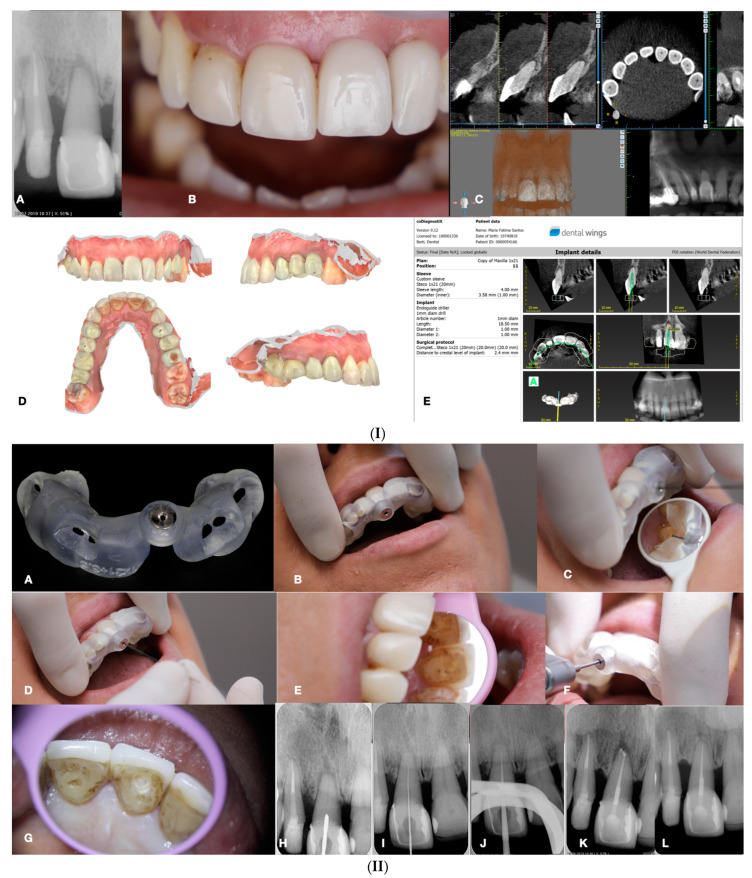
(**I**) (**A**) Preoperative periapical radiograph of calcified maxillary central incisor diagnoses with apical periodontitis. (**B**) Preoperative clinical buccal view of the maxillary central incisors. (**C**) Preoperative cbct scanning of the maxilla. (**D**) Preoperative intraoral scanning of the maxilla. (**E**) Matching the CBCT scanning with the intraoral scanning and designing the guide in the codiagnostiX 10.5 software. (**II**) (**A**) Three-dimensional printed surgical guide with lateral windows to check the adaptation. (**B**) Intraoral fitting of the surgical guide. (**C**,**D**) Pencil marking of the access location point lingual and buccal view. (**E**) Access point mark to remove the enamel. (**F**) Fitting of the surgical guide and drilling the initial pilot hole. (**G**) Clinical palatal view of the guided access. (**H**) Intraoperative radiograph of the pilot drill (Steko bur). (**I**,**J**) Length determination and gutta-percha fitting radiographs. (**K**) Postoperative radiograph. (**L**) Two-year follow-up radiograph reveals healing (courtesy of Dr. Hugo Sousa Dias).

The static-guided technique involves the utilization of a guiding template in conjunction with cone beam computed tomography (CBCT) to aid in the localization of severely calcified root canals (Figure 15) [20,44,47,48,50]. This technique relies on the design of the guide, which is based on the anatomical features of the root canal and the surrounding tooth structures, obtained through CBCT images and either an impression or intra-oral surface scan, respectively (Figure 15) [51]. 

Specialized software, such as coDiagnostiX 10.5 (Dental Wings Inc., Montreal, Canada), is employed to superimpose CBCT data and 3D intra-oral scans, facilitating virtual planning of the access cavity [47,51]. Subsequently, a 3D virtual template is generated to produce the physical model of the endodontic guide, which guides the bur into the calcified root canal [47,51]. Static-guided access in cases of pulp canal obliteration (PCO) in anterior teeth has been previously documented in the literature and is described as a safe and predictable technique for minimally invasive access to calcified canals. This approach aids in preserving tooth structure, avoiding technical errors, and improving long-term prognosis [48,52]. Krastl et al. [53] were pioneers in describing the static-guided technique in vivo, specifically on a maxillary central incisor with PCO and apical periodontitis. Originally developed for implantology, this technology has been adapted for use in endodontics, surgery, and conventional access [54]. The static-guided technique involves accessing and locating root canals through a guiding template created via tomographic planning [48,52]. The guiding template sleeves direct the position of the access burs, enhancing perforation precision during access and ensuring adequate tomographic planning (see Figure 15). Ex vivo studies have demonstrated the high accuracy of the guided endodontics technique. Buchgreitz et al. [55] concluded that the mean distance between the drill path and the target was less than 0.7 mm, while Zehnder et al. [43] showed low deviations between planned and prepared access cavities, with mean angle deviations of 1.81° [56]. The accuracy of guided splints depends on various factors, including the type of support and study, technique used to produce the template, planning software, discrepancy between the drill and cylinder guide, degree of wear of the drill, and number of guides used [56]. However, guided templates are associated with limitations, such as inaccuracy, high economic cost, long therapeutic time, and potential complications [56].

Inaccuracies in guided techniques are partly attributed to the loose fit between the drill and the sleeve, which is necessary to prevent heat development during preparation [44,55]. Studies have shown improvements in accuracy by optimizing the fit between the bur and the sleeve, with the use of a metal sleeve recommended for better control of the drill [57,58]. Careful irrigation during drilling is also essential to avoid heat-related injuries to the periodontal ligament and adjacent bone [47,50]. The static-guided technique has demonstrated sufficient accuracy to establish a safe treatment method for teeth with PCO, with no significant differences observed between operators. However, it is important to note that this technique has anatomical limitations, particularly in severely curved canals and cases with radicular grooves, isthmuses, and oval roots. Additionally, limited inter-occlusal distance and the requirement for additional instrument length pose challenges, making the technique contraindicated in cases of curved canals and limited mouth opening [54,55]. While conventional root canal treatment (RCT) and apical surgery are alternative options for teeth with PCO, they have their limitations, including prolonged treatment times and increased risk of iatrogenic errors. Access guides are manufactured by overlapping CBCT data with intra-oral scans of the target area, with CBCT playing a crucial role in preoperative visualization and treatment planning [48,59]. Despite the higher radiation doses associated with CBCT, its use has contributed to increasing the success rate of endodontic treatments by optimizing preoperative planning [48]. The planning process of the static-guided technique is time-consuming compared to conventional RCT due to the need for CBCT acquisition, intra-oral scanning, virtual planning, and template fabrication. However, the reduced chair time and potential reduction in iatrogenic errors justify the additional costs associated with this technique [53,57]. Rubber dam isolation is essential for the success of endodontic treatment, although initial access without a rubber dam may be necessary for adapting the guide [58,60,61]. Despite its advantages, the static-guided technique has limitations, including restricted visualization during treatment and the intermittent removal of the guide to ensure proper path following. To address these limitations, the dynamic-guided technique presents an alternative approach worth considering.

## 11. Dynamic-Guided Technique

The dynamic-guided technique employs a mobile unit equipped with an overhead light, a stereoscopic motion-tracking camera, and a computer with planning software to guide a calibrated handpiece in real time. Motion tracking technology allows the system to monitor the position of both the patient and the dental handpiece throughout the procedure. The ideal drill position is planned virtually by the surgeon using CBCT data uploaded into the planning software. Sensors attached to the surgical handpiece and the patient’s head or teeth transfer 3D spatial information to a stereo tracker [62,63]. Experimental studies have confirmed the accuracy of the dynamic navigation technique, highlighting advantages over traditional methods, such as complexity, skill dependency, and time consumption [58,62,64,65]. Similar to the static navigation approach, the dynamic navigation technique begins with a high-resolution preoperative CBCT scan to plan the entry point, pathway, depth, and angle of the bur. The operator can visually monitor the bur’s progression on a laptop screen in real time, with depth indicated by color changes on the depth gauge [64]. One of the primary benefits of dynamic navigation is its direct view of the operatory field, allowing clinicians to adjust the direction of the bur in real time. Unlike static guidance, dynamic navigation does not require an intra-oral scan and is particularly useful in cases of limited mouth opening or posterior region treatments where a template may be impractical [64]. Additionally, dynamic navigation enables the treatment of endodontic urgencies without the need for additional template design and printing [58,63,64]. However, the high acquisition cost and the need for operator proficiency are significant disadvantages of dynamic navigation. Unlike static guidance, dynamic navigation requires practice to prepare precise access cavities, as operators must adjust to looking at a monitor rather than directly at the patient. Some commercially available systems are bulky and may pose practical challenges in clinical settings [63,66].

## 12. Conclusions and Future Perspectives

The use of new technologies, the knowledge of pulp anatomy and the calcification process, and the interpretation of radiographic exams are the keys to achieving success in the treatment of calcified canals [67]. Guided endodontics using static or dynamic navigation appears to be a safe and minimally invasive method for detecting and negotiating calcified root canals [65]. However, despite the excitement of managing the clinical problem via such technological advancements, the question must be asked as to whether such sophisticated methods are necessary. If CBCT alone can be used to identify the canal and allow the dentist to prepare access to the correct depth, then it may be possible to dispense expensive accouterments such as stents [25]. Almost 90% of the calcified canals can be negotiated to the apical third with the help of conventional techniques and the operating microscope [68]. The success rate after a follow-up period of 3 years was found to be 80 % [68]. Achieving technical patency to the apical third is the most important intraoperative treatment factor associated with a successful outcome in teeth with apical periodontitis [69]. In calcified root canal systems, achieving technical patency to the apical third can be feasible and predictable [68]. The visual feedback provided by a dental operating microscope (DOM) [70] through coaxial illumination, magnification, and stereoscopic depth perception coupled with the information provided by a high-resolution CBCT imaging examination might be adequate to solve most calcified canal negotiation cases.
